# Hypertensive Retinopathy among Patients with Hypertension Attending the Department of Ophthalmology in a Tertiary Care Centre: A Descriptive Cross-sectional Study

**DOI:** 10.31729/jnma.8213

**Published:** 2023-07-30

**Authors:** Pradeep Bastola, Sheeksha Bastola

**Affiliations:** 1 Department of Ophthalmology, Chirayu National Hospital, Basundhara, Kathmandu, Nepal; 2 Gandaki Medical College Teaching Hospital, Pokhara, Kaski, Nepal

**Keywords:** *biomarkers*, *body mass index*, *cardiovascular diseases*, *hypertensive retinopathy*, *visual acuity*

## Abstract

**Introduction:**

Hypertension is one of the major cardiovascular diseases leading to disability or death. Persistently elevated blood pressure leads to retinal vascular changes, also known as hypertensive retinopathy. The aim of this study was to find out the prevalence of hypertensive retinopathy among patients with hypertension attending the Department of Ophthalmology in a tertiary care centre.

**Method:**

A descriptive cross-sectional study was conducted among hypertensives patients presenting to the Department of Ophthalmology in a tertiary care centre from 1 June 2022 to 1 February 2023 after receiving ethical approval from the Institutional Review Committee (Reference number: CMC-IRC 078/079-228). Those patients who did not provide informed consent and patients with chronic debilitating systemic conditions, patients encountered in emergency wards, and hypertensive patients below 15 years of age and above 75 years of age were excluded from the study. Convenience sampling was done. Point estimate and 95% Confidence Interval were calculated.

**Results:**

Among 336 study participants, hypertensive retinopathy was found in 66 (19.64%) (15.39-23.89, 95% Confidence Interval) study subjects in at least one eye.

**Conclusions:**

The prevalence of hypertensive retinopathy was lower in our study when compared to other studies.

## INTRODUCTION

Hypertension affects approximately 50 million individuals in the United States and approximately one billion worldwide. A spectrum of vascular changes can be seen in the retinal vasculature of patients with hypertension and these changes are called hypertensive retinopathy (HR).^1^-^2^

The seventh report of the Joint National Committee on prevention-detection-evaluation- and treatment of high blood pressure recommends the evaluation of HR as a part of standard care of hypertension and lists HR as a marker of target organ damage.^2^ Prolonged uncontrolled systemic hypertension may result in visual impairment or blindness. It is known that hypertension aggravates atherosclerosis and vice versa.^3^'^5^

This study aimed to find out the prevalence of hypertensive retinopathy among patients with hypertension attending the Department of Ophthalmology in a tertiary care centre.

## METHODS

A descriptive cross'sectional study was conducted on the patients presenting to the Outpatient Department (OPD) of Ophthalmology at Chitwan Medical College Teaching Hospital, Bharatpur, Chitwan, Nepal after obtaining ethical clearance from Institutional Review Committee (Reference number: CMC-1RC/078/079-228). The study was conducted from 1 June 2022 to 1 February 2023. All hypertensive patients visiting the Department of Ophthalmology and referred cases from the Department of Medicine willing to get enrolled in the study were included in the study. The patients who were not willing, who did not provide informed consent, patients with chronic debilitating systemic conditions, patients encountered in emergency wards, and hypertensive patients below 15 years of age and above 75 years of age were excluded from the study. A convenience sampling technique was used. The sample size was calculated using the following formula:


$$\begin{array}{ll} \text{n} & ={\text{Z}}^{2}\times \frac{\text{p}\times \text{q}}{{\text{e}}^{\text{2}}}\\ & =1.96^{2}\times \frac{0.50\times 0.50}{0.05^{2}}\\ & =267 \end{array}$$

Where,

n = minimum required sample size
Z = 1.96 at 95% Confidence Interval (CI)
p = prevalence taken as 50% for maximum sample size calculation
q = 1-p
e = margin of error, 6%

The required sample size was 267. However, we took 336 participants.

After explaining the purpose of the study and the confidentiality of data collection, informed consent was obtained from each participant. The study participants were evaluated in detail in the following sequence: visual acuity measurement of each eye separately (unaided and with a pinhole), extraocular movement assessment, cover test, cover-uncover test, refraction using a heine beta 200 retinoscope, anterior segment examination with a slit lamp, and detailed fundus examination. Fundus evaluation was done using direct ophthalmoscope and indirect ophthalmoscope using +20 Dioptre (D), +78D, and +90D lenses. Tropicamide 1% or tropicamide 0.8% and/or phenylephrine 5% eye drops were used to dilate the pupils for fundus evaluation. All four quadrants of the retina-superior, inferior, nasal, and temporal were examined in detail. The macular and foveal region was given special attention during the fundus evaluation. The significant findings from the fundus were documented and a picture of the fundus was drawn only when positive findings were observed. Keith Wagner Barker and Wong Mitchell's classification and grading were used for hypertensive retinopathy.^4,6^ The atherogenic index of plasma (AIP) was calculated using a formula.^7^

Data were entered and analysed using IBM SPSS Statistics version 26.0. Point estimate at 95% CI was calculated.

## RESULTS

Among 336 participants, 66 (19.64%) (15.39-23.89, 95% CI) study subjects had hypertensive retinopathy changes in at least one eye. Grade I hypertensive retinopathy was the commonest accounting for 48 (72.72%) followed by Grade II HR in 8 (12.12%), Grade IV HR in seven (10.6%), and Grade III HR in 3 (4.54%) in the study subjects (Table 1).

**Table 1:** Grades of hypertensive retinopathy (n= 66).

Variable	n (%)
Grade I	48 (72)
Grade II	8 (12.12)
Grade III	3 (4.54)
Grade IV	7 (10.60)

Forty-eight (72.72%) of study subjects were at a modest future risk of having stroke, sub-clinical stroke, cognitive impairment, chronic heart disease (CHD), and death.

Among 66 patients, 24 (36.36%) study subjects were diagnosed hypertensive for less than five years. Visual acuity of the study subjects was impaired in 13 (19.69%). Relative afferent pupillary defect and afferent pupillary defect were seen in 1 (1.51%) of the study subjects (Table 2).

**Table 2:** Clinical characteristics of the patients with hypertensive retinopathy (n= 66).

Variables	n (%)
**Hypertensive, and treatment status**	
Hypertension less than 5 years	24 (36.36)
Hypertensive for more than 5 years	23 (34.84)
Hypertensive under medication but not under control	7 (10.60)
Recently diagnosed hypertension on lifestyle modification	12 (18.18)
**Body mass index (BMI)**	
Normal	22 (33.33)
Overweight	20 (30.30)
Obese	24 (36.36)
**Electrocardiogram (ECG) findings**	
Sinus rhythm	49 (74.24)
Ischemic heart disease (current or past)	6 (9.09)
Left ventricular dysfunction	9 (13.63)
Poor (R) wave progression	1 (1.51)
Left ventricular hypertrophy	1 (1.51)
**AIP**	
Normal	-
Moderately increased AIP	1 (1.51)
High AIP	65 (98.48)
**Presenting visual acuity with the best possible correction**	
Normal	53 (80.30)
Impaired	13 (19.69)
**The pupillary reaction**	
Normal size, normal reaction	63 (95.45)
Relative afferent pupillary defect (RAPD)	2 (3.03)
Afferent pupillary defect (APD)	1 (1.51)

Males 36 (54.54%) just outnumbered the females. Urban patients formed the majority of the study population accounting for 50 (75.75%) (Table 3).

**Table 3:** Demographic profile of the patients with hypertensive retinopathy (n= 66).

Variables	n (%)
**Gender distribution**	
Male	36 (54.54)
Female	30 (45.45)
**Geographical representation**	
Urban	50 (75.75)
Rural	16 (24.24)
**Occupation wise distribution**	
Farmer	14 (21.21)
Office work	18 (27.27)
Retired	6 (9.09)
Housewife	8 (12.12)
Teacher	11 (16.66)
Business	9 (13.63)

## DISCUSSION

In our study, among 336 patients, 66 (19.64%) patients had hypertensive retinopathy in at least one eye. Grade I hypertensive retinopathy was the commonest accounting for 48 (72.72%) followed by Grade II HR in 8 (12.12%), Grade IV HR in 7 (10.6%), and Grade III HR in 3 (4.54%) in the patients. The prevalence of HR in our study was low when compared to similar studies from Nepal, and Iran. In their study, the overall prevalence of HR was 54%, 39.90%, and 38.95% respectively.^3,8,9^ However, in a recent study from central Nepal the prevalence of hypertensive retinopathy of all grades, was just 10.72%, which was lower than the estimate from our study.^1^ This variability in the prevalence of HR within a similar context could be due to different sampling techniques, sample sizes, and diagnostic methods. The very low prevalence of HR 10.72%, in a study from Nepal,^1^ could be hugely contributed to the COVID-19 disease and lockdown as the study was conducted during that period. The lower prevalence of HR in the current study in normal times could be attributed to the increased health-seeking behavior of the patients, compliance, and adherence to the treatment, health education, and health promotion.

HR has been used as a measure to assess cardiovascular accidents in several studies across the globe, and the finding from those studies compared with our study findings.^10,11^ In a study from the Netherlands, it was suggested that grading hypertensive retinopathy in hypertensives and using HR as a tool was inconsistent, and not suitable as they concluded that, the association of retinopathy with other predictive indicators for organ damage is inconsistent and its association with cardiovascular complications is weak besides a huge array of interobserver variations to grade HR during examinations.^12^

Of all the study subjects, 24 (36.36%) study subjects were diagnosed hypertensive for less than 5 years, whereas 23 (34.84%) study subjects were hypertensive for more than 5 years, 12 (18.18%) study participants were on lifestyle modification, only seven (10.60%) of the study subjects had uncontrolled hypertension despite treatment. Forty-eight (66.69%) study subjects were either overweight or obese. These study findings of the current study were comparable to studies from Nepal.^1,3,9^ Atherogenic index of plasma (AIP) is an excellent biomarker to predict cardiovascular accidents, in the current study raised AIP in all the study subjects pointed towards abnormal lipid levels in the study subjects with HR and added future risk of cardiovascular accidents. This finding of our study correlated and compared well with a study from Nepal.^1^ In the present study, 17 (25.76%) study subjects had some kind of ECG abnormality of which the patients were not aware, this finding from the current study compared well with studies from Nepal, that in HR patients ECG changes can occur and are strong predictors of cardiovascular accidents both past and future.^1,3^ Visual impairment in 13 (19.69%), and the presence of a relative afferent pupillary defect or afferent pupillary defect in three (4.55%) study subjects with HR was attributed to chronic grade IV hypertensive retinopathy and consecutive optic atrophy in patients with uncontrolled hypertension (HTN). The ECG abnormality, and visual impairment in the study participants did indicate target organ damage, and hinted towards further risks in the future, this finding from our study correlated very well with findings from a study done elsewhere.^13^

Males outnumbered the females. Urban patients formed the majority of the study population. Occupation-wise office work 18 (27.27%) followed by farming 14 (21.21%), and teaching 11 (16.66%) were commoner in the study subjects with hypertensive retinopathy. The demographic profile of our study population was consistent with the studies from Nepal.^1,3,9^

The current study emphasized the fact that routine ophthalmological checkup of hypertensive patients is a must; particularly given the detection of hypertensive retinopathy in our setting. We would suggest using the risk prediction classification as used in this current study for all HR patients to prevent morbidity and mortality caused by cardiovascular accidents. Patients having signs of HR require a further thorough evaluation of cardiovascular risk factors, which also includes lipid profile as early identification and treatment of these risk factors may help prevent blindness as well as cardiovascular morbidity and mortality. The atherogenic index of plasma (AIP) can be a helpful biomarker to predict future cardiac morbidities in patients with HR.

Our study was based on single hospital with a small sample size conducted within a limited time period. Therefore, it cannot be generalised to the broader prospect.

## CONCLUSIONS

The prevalence of hypertensive retinopathy was found to be lower than other studies done in similar settings.

Hypertensive retinopathy can be a tool in predicting future cardiovascular accidents and should be widely used in countries like Nepal to combat premature deaths due to non-communicable diseases (NCDs), this would be achieved by a strong referral pathway in all the hospitals in Nepal. An ophthalmologist plays a vital role to screen and detect hypertensive retinopathy in all individuals with hypertension, besides recommending a further plan of management.
